# Multivariate Brain Functional Connectivity Through Regularized Estimators

**DOI:** 10.3389/fnins.2020.569540

**Published:** 2020-12-08

**Authors:** Raymond Salvador, Norma Verdolini, Beatriz Garcia-Ruiz, Esther Jiménez, Salvador Sarró, Elisabet Vilella, Eduard Vieta, Erick Jorge Canales-Rodríguez, Edith Pomarol-Clotet, Aristotle N. Voineskos

**Affiliations:** ^1^FIDMAG Germanes Hospitalàries Research Foundation, Barcelona, Spain; ^2^Centro de Investigación Biomédica en Red de Salud Mental (CIBERSAM), Madrid, Spain; ^3^Bipolar and Depressive Disorders Unit, Institute of Neuroscience, Hospital Clinic, University of Barcelona, IDIBAPS, Barcelona, Spain; ^4^Hospital Universitari Institut Pere Mata, IISPV, Universitat Rovira i Virgili, Tarragona, Spain; ^5^Campbell Family Mental Health Research Institute, Toronto, ON, Canada; ^6^Department of Psychiatry, University of Toronto, Toronto, ON, Canada

**Keywords:** brain connectivity, ridge regression, random forest, global brain connectivity, age, gender

## Abstract

Functional connectivity analyses are typically based on matrices containing bivariate measures of covariability, such as correlations. Although this has been a fruitful approach, it may not be the optimal strategy to fully explore the complex associations underlying brain activity. Here, we propose extending connectivity to multivariate functions relating to the temporal dynamics of a region with the rest of the brain. The main technical challenges of such an approach are multidimensionality and its associated risk of overfitting or even the non-uniqueness of model solutions. To minimize these risks, and as an alternative to the more common dimensionality reduction methods, we propose using two regularized multivariate connectivity models. On the one hand, simple linear functions of all brain nodes were fitted with ridge regression. On the other hand, a more flexible approach to avoid linearity and additivity assumptions was implemented through random forest regression. Similarities and differences between both methods and with simple averages of bivariate correlations (i.e., weighted global brain connectivity) were evaluated on a resting state sample of *N* = 173 healthy subjects. Results revealed distinct connectivity patterns from the two proposed methods, which were especially relevant in the age-related analyses where both ridge and random forest regressions showed significant patterns of age-related disconnection, almost completely absent from the much less sensitive global brain connectivity maps. On the other hand, the greater flexibility provided by the random forest algorithm allowed detecting sex-specific differences. The generic framework of multivariate connectivity implemented here may be easily extended to other types of regularized models.

## Introduction

Matrices based on correlations or similar bivariate measures have frequently been the starting point of many functional connectivity analyses (Raichle, 2011; Bassett and Bullmore, 2017). Although this has been a fruitful approach, reliance on bivariate associations may not be the optimal strategy to fully explore the complex relations underlying brain activity. Instead, a multivariate approach considering signals from different regions of the brain seems more adequate for this purpose.

However, such an approach is not without its technical challenges, which include above all those related to the high dimensionality of imaging data. In the frequent scenario where the number of brain regions (N) is similar to the number of available time points (p), overfitting is a risk. Also, if voxels are considered instead of regions (i.e., the *N* >> p situation), the problem of multiplicity of solutions will occur (Hastie et al., 2009). Although these problems have traditionally been dealt with dimension reduction method such as the principal component analysis, independent component analysis, and partial least squares (Calhoun et al., 2009; McIntosh and Misic, 2013; Salvador et al., 2017), there is also the alternative of applying model regularization.

When regularizing, a model including the full set of original variables is fit by imposing one or more constraints on the values of parameter estimates (Hastie et al., 2009). Usually, regularization will involve choosing a specific shape for the function relating the dependent variable with all independent variables in the model. However, finding the optimal shape for this function in a high dimensional space may be challenging, and this is usually avoided by assuming a simple linear model, as in ridge or lasso regressions, or by selecting a specific non-linear shape through non-linear kernels, as it is done when using support vector machines (Cortes and Vapnik, 1995). Still, any of these alternatives have a good chance of missing the optimum shape for that function. This limitation may be overcome by considering alternatives such as random forest (RF) regression or feed-forward neural networks, which do not require setting a specific function shape (Hastie et al., 2009; Goodfellow et al., 2016).

Here, we propose extending functional connectivity analyses by fitting multivariate functions that relate the temporal dynamics of a region with the rest of the brain. This is carried out by means of two different regularization methods. As a first option, ridge regression is applied, and as a second option, RF regression, which allows relaxing both linearity and additivity assumptions held by the ridge model, is also considered. The information contained in connectivity maps generated by both regularization methods is evaluated in a resting-state functional magnetic resonance imaging (fMRI) sample of *N* = 173 healthy individuals. Specifically, connectivity patterns related to sex and age are explored using both methods and compared with those derived from maps of averaged bivariate correlations, also known as weighted global brain connectivity (GBC) maps (Cole et al., 2010).

## Materials and Methods

### General Framework

As a general framework, we propose modeling the functional coupling between the temporal dynamics of each region *i* and the remaining (*N*-1) regions of the brain through a generic function

(1) $$Y_{i}=f\,\left(Y_{1},\ldots ,Y_{i-1},Y_{i+1},\ldots ,Y_{N}\right)+\varepsilon_{i}$$

in which the degree of coupling can be simply quantified by

(2) $$C\,o\,r\,\left(Y_{i},f\,\left(Y_{1},\ldots ,Y_{i-1},Y_{i+1},\ldots ,Y_{N}\right)\right)$$

i.e., the correlation between the actual values of the time points of region *i* (*Y*_*i*_) and the values provided by the multivariate connectivity function ($\overset{\wedge }{Y_{i}}$).

### Ridge Regression Connectivity Maps

As a simple option, we may consider *f* to be a linear additive model (i.e., a multiple regression model)

(3) $${\overset{\wedge }{Y}}_{i}=\beta_{0}+\beta_{1}Y_{1}+\ldots +\beta_{i-1}Y_{i-1}+\beta_{i+1}Y_{i+1}+\ldots +\beta_{N}Y_{N}$$

where *Cor*(*Y_i_*, $\overset{\wedge }{Y_{i}}$) (Eq. 2) will quantify the degree to which the temporal dynamics of *i* and the remaining brain regions are linearly related. However, fitting Eq. 3 to fMRI data using standard methods (i.e., ordinary least squares) will, if even possible, lead to unreliable estimates, as N (the number of regions) will be similar or even larger than the number of available time points (p), causing either overfitting or leading to the non-uniqueness of solutions.

**FIGURE 1:**
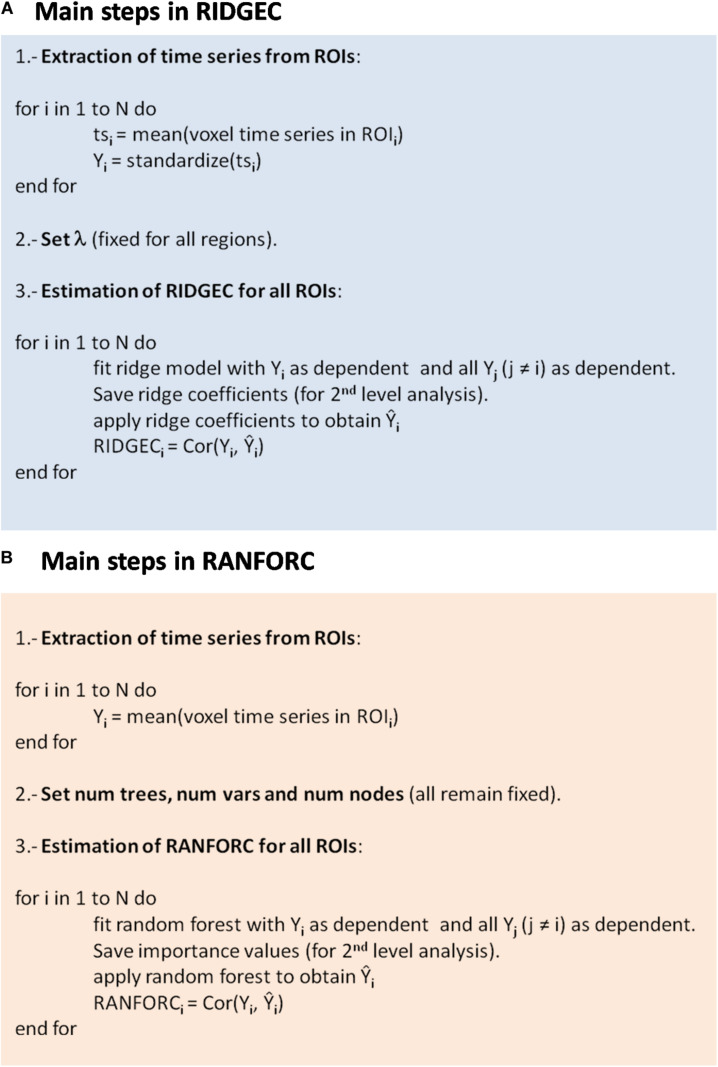
Workflow listing the major steps required to calculate **(A)** ridge based connectivity values and **(B)** random forest connectivity values for each one of the N regions extracted from an fMRI preprocessed dataset. Saving ridge regression coefficients and importance values are optional.

Such limitations, however, can be easily overcome by setting a restriction on the parameter estimates (i.e., regularizing). Specifically, ridge regression imposes the following restriction

(4) $$\sum_{i=1}^{N}\beta_{i}^{2}<c\,t$$

which makes the least-squares minimization a constrained problem regulated by a Lagrange multiplier (λ ≥ 0). Selecting an adequate value for λ will be important to achieve a good balance between bias and variance (i.e., to find a model that avoids overfitting, but it is not too constrained) (James et al., 2013). The optimal value for λ will depend, among other aspects, on both the number of brain regions and the number of available time points. Crucially, once chosen, λ should remain constant through all fittings of Eq. 3 if comparable connectivity estimates are wanted between all regions of the brain and between all individuals of a study. In addition, for Eq. 4 to be meaningful, all time series will have to be previously rescaled to unit variance. A summary of the major steps required to apply ridge regression connectivity (RIDGEC) to an fMRI dataset is given in Figure 1A.

### Random Forest Connectivity Maps

The assumptions of additivity and linearity of Eq. 3 may be relaxed by considering non-linear relations between brain regions. To avoid having to choose a specific shape for Eq. 1, one can consider other approaches that do not require specifying such function in an explicit way. One option is using tree-based methods, which stratify the predictor space into a number of discrete regions when fitting the regression model (Ho, 1995). Furthermore, results from fitting different regression trees may be combined in what is known as a random forest to obtain more reliable estimates. As with ridge regression, RF regression will deliver estimates of values for region *i* ($\overset{\wedge }{Y_{i}}$) based on all other regions in the brain.

(5) $${\overset{\wedge }{Y}}_{i}=RF\left(Y_{1},\ldots ,Y_{i-1},Y_{i+1},\ldots ,Y_{N}\right)$$

and again, the strength of connectivity will be quantified by Eq. 2 [*Cor*(*Y_i_*, $\overset{\wedge }{Y_{i}}$)].

Like any other multivariate regression method, RFs are also vulnerable to overfitting. For them, regularization is achieved by averaging results from many different trees, by restricting the number of independent variables considered in each tree, and by allowing for a moderate amount of branching (number of nodes in each tree) (Hastie et al., 2009). Critically, like λ in ridge, once all these quantities are set, they should remain fixed to be able to have comparable connectivity values for the different regions and individuals. Figure 1B describes the major steps involved in applying random forest connectivity (RANFORC) to an fMRI dataset.

### Getting Further Insight With Ridge Coefficients and Variable Importance

Although both RIDGEC and RANFORC provide a single multivariate connectivity score for each region *i*, fittings of their respective models also supply exhaustive information on the relevance of the different regions in each multivariate measure, which is something that may bring further insights on the nature of connectivity patterns observed in a given sample (second-level analysis). For the RIDGEC, this information is directly provided by the magnitude of the regression coefficients (betas) in the fitted model of Eq. 3. For the RANFORC there are no coefficients directly available, but an estimate of the relevance of each variable may be obtained by quantifying the reduction in the sum of squares of the error attributable to that variable, a quantity that is known as variable importance (Hastie et al., 2009). As shown in Figure 1, saving coefficients and variable importances is a choice that will depend on the interest in carrying out second-level analyses.

### Resting-State Functional Magnetic Resonance Imaging Dataset

To explore the properties of both RIDGEC and RANFORC algorithms, they were applied to a sample of 173 healthy individuals scanned at rest in a 3.0-T Philips Ingenia machine. Table 1 provides summary statistics on age and sex for the participants. All subjects gave written informed consent before participation. All the study procedures had been previously approved by the Comité de Ética de la Investigación de FIDMAG Hermanas Hospitalarias and adhered to the Declaration of Helsinki.

**TABLE 1:** Summary data on sex and age for the whole sample and for the age-matched sample used to evaluate sex-specific connectivity patterns.

	*N*	Sex	Age (all)	Age (Females)	Age (Males)	*T*-test
Whole Sample	173	110 Females (63.5%) 63 Males (36.4%)	Mean = 40.42 SD = 10.37 Range = 18–61	Mean = 42.43 SD = 9.48 Range = 22–61	Mean = 36.92 SD = 10.99 Range = 18–59	*t* = 3.467, df = 171, *p* = 0.0007
Age-Matched Sample	126	63 Females (50.0%) 63 Males (50.0%)	Mean = 37.63 SD = 10.14 Range = 18–60	Mean = 38.33 SD = 9.24 Range = 22–60	Mean = 36.92 SD = 10.99 Range = 18 – 59	*t* = 0.781 df = 124, *p* = 0.436

*Last column reports results from a *t*-test comparing mean age between both sexes.*

Parameters for the resting fMRI bold sequence were: repetition time = 2s, echo time = 30 ms, flip angle = 70°, inplane resolution = 2.5 × 2.5 mm, slice thickness = 2.5, number of slices = 54, and number of volumes = 256, which led to a total scan time of 8 min and 32 s. Parameters for the T1 structural images were: repetition time = 1 ms, echo time = 0.46 ms, flip angle = 8°, inplane resolution = 1 × 1 mm, and slice thickness = 1 mm. fMRI preprocessing steps included movement correction, spike scrubbing, regression of noise-independent components, non-linear normalization to the Montreal Neurological Institute space, regression of noise from ventricles and white matter, and low-frequency filtering in the 0.1–0.02-Hz interval (Salvador et al., 2017). Specifically, for the regression of noise-independent components, individual independent component analyses were previously run with Melodic, a module included in FSL (Smith et al., 2004), and those components showing clear noise patterns (most frequently edge effects due to movement) were selected. Time series of the selected components were regressed out from the time series of each voxel, and residuals were kept as the denoised time series. Once preprocessed, mean time series were extracted from the Brainnetome atlas, which include 246 cortical and subcortical regions of interest (ROIs) (Fan et al., 2016).

### Processing of Individual Connectivity Maps

Ridge regressions were carried out with functions contained in the glmnet R library (Friedman et al., 2010), and the randomForest R library (James et al., 2013) was used for RF regressions. For the initial selection of the regularization parameters, we run exploratory analyses in a single individual using a wide range of possible values, and we selected a set of values that neither overfit (did not lead to correlations too close to 1) nor led to strong biases (too small correlations). Once selected, these parameters were kept constant through all fittings involving the different ROIs and individuals. Specifically, a value of λ = 10 was used for the ridge, and 1,000 trees with 10 variables each and a maximum of four nodes per tree were used for the RFs. For the individual GBC analyses, averages of the absolute values of bivariate correlations were considered, but, additionally, GBC maps based on the previous thresholding of correlations were also calculated to assess the robustness of GBC results. With that aim, four thresholds were applied: cor > 0, cor > 0.1, cor > 0.2, and cor > 0.3. All connectivity measures were Fisher transformed before carrying out the group analyses.

### Group Averaged, Sex and Age-Related Connectivity Patterns

Group average RIDGEC, RANFORC, and GBC maps, sex-specific maps, and age-related connectivity maps were derived from the individual connectivity images.

For the analysis of gender related connectivity patterns, an age matched subsample of 63 males and 63 females was used and *t*-tests were performed to look for gender specific differences (see Table 1). For the analysis of age related patterns, regression models were fit to the original sample of 173 subjects and gender was included as covariate.

Additionally, in order to see if patterns observed could be related to structural abnormalities, a new set of models was fit for both age and gender taking grey matter partial volumes as covariates. To do so, grey matter partial volume maps were derived from individual T1 structural images by means of the FAST module included in the FSL software (Smith et al., 2004). These maps were then normalized to the MNI space, spatially filtered with a gaussian filter (sigma = 3 mm) and mean values from the Brainnetome parcellation were extracted. In all analyses a False Discovery Rate (FDR) correction was applied to account for multiple comparisons.

## Results

### Average Connectivity Patterns

**FIGURE 2:**
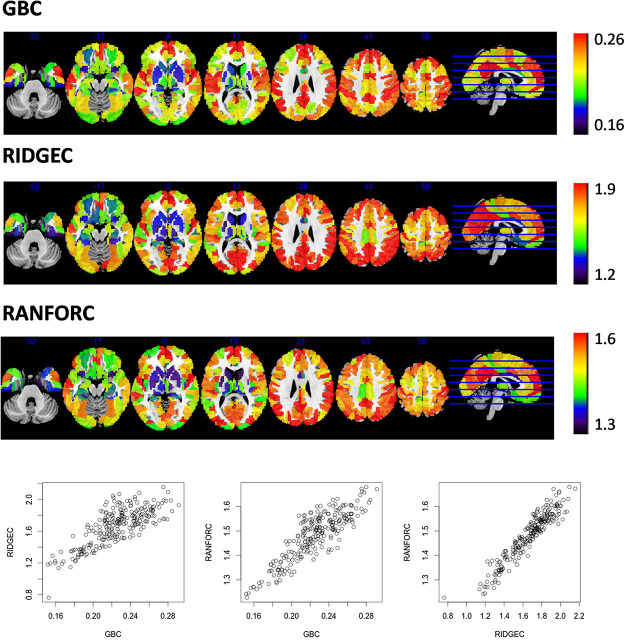
Maps of averages from GBC, RIDGEC, and RANFOR for the whole sample of 173 healthy individuals. Scatterplots in the bottom show, for each one of the 246 regions of the Brainnetome atlas, their group averages in each possible pair of brain maps. All values were previously Fisher transformed into z-scores.

Group average RIDGEC, RANFORC, and GBC maps are shown in Figure 2. As it can be appreciated from the figure, there are clear commonalities among the three maps, including the low connectivity levels in ventral and subcortical structures and the high connectivity values in posterior cingulate and medial frontal structures. However, as it can also be appreciated from the scatterplots in this figure, although there is a clear linear relation between RIDGEC and GBC and between RANFORC and GBC, both regularized maps also contain differential connectivity patterns not provided by the averages of bivariate correlations.

**FIGURE 3:**
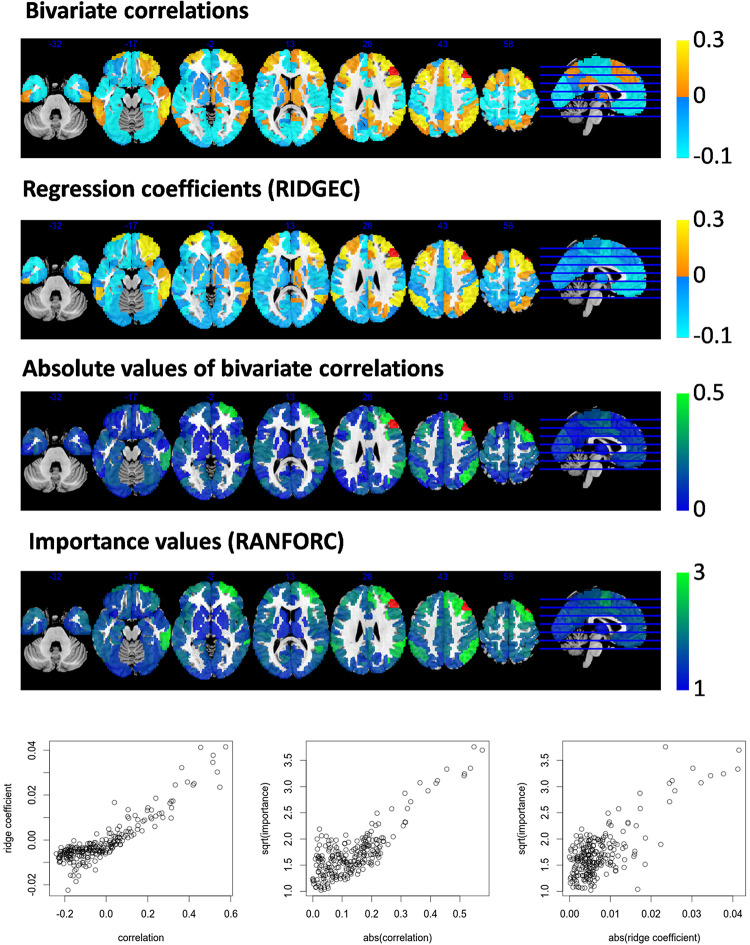
Taking area A8vl_R of the Brainnetome atlas (located in the right dorsolateral prefrontal cortex and shown here in red) as the target area for the RIDGEC and RANFOR fittings, the group averaged maps of ridge regression coefficients and variable importances are shown together with the bivariate correlation map (i.e., derived from a standard seed-based correlation analysis). Scatterplots in the lower part of the figure show, for each region of the template, its group averaged values. Absolute values of correlations and ridge coefficients are taken to make them comparable with importances (as the latter are always positive).

**FIGURE 4:**
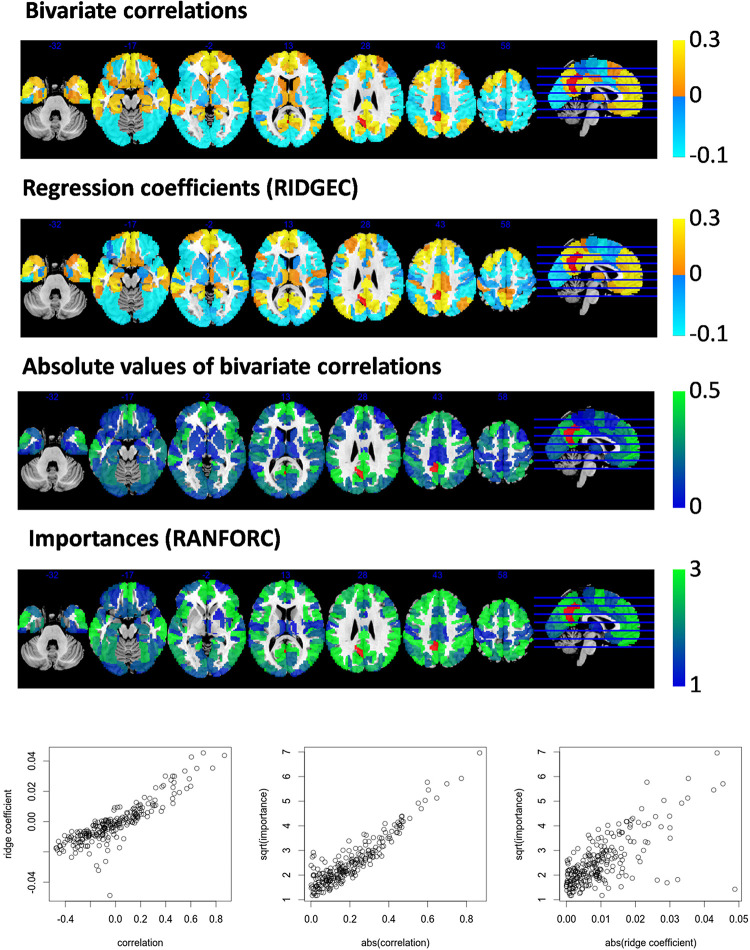
Taking area A31_L of the Brainnetome atlas (located in the left posterior cingulate cortex and shown here in red) as the target area for the RIDGEC and RANFOR fittings, the group averaged maps of ridge regression coefficients and variable importances are shown together with the standard bivariate correlation map. Scatterplots in the lower part of the figure show, for each region of the template, its group averaged values. Absolute values of correlations and ridge coefficients are taken to make them comparable with importances (as the latter are always positive).

Further similarities and differences between the newly proposed maps and the information conveyed by simple bivariate correlations can be appreciated from second-level analyses. As examples, values from ridge regression coefficients and variable importances are compared with bivariate correlations in models fit for an ROI in the right dorsolateral prefrontal cortex (Figure 3) and an ROI in the left posterior cingulate (Figure 4).

From both Figures 3, 4, it is clear that, as it was previously observed for RIDGEC and RANFORC scores in Figure 2, there are evident similarities between patterns in bivariate correlations, ridge regression coefficients, and variance importance scores. Still, scatterplots in both figures show that these three measures of association differ to some degree.

### Sex-Related Connectivity Patterns

**FIGURE 5:**
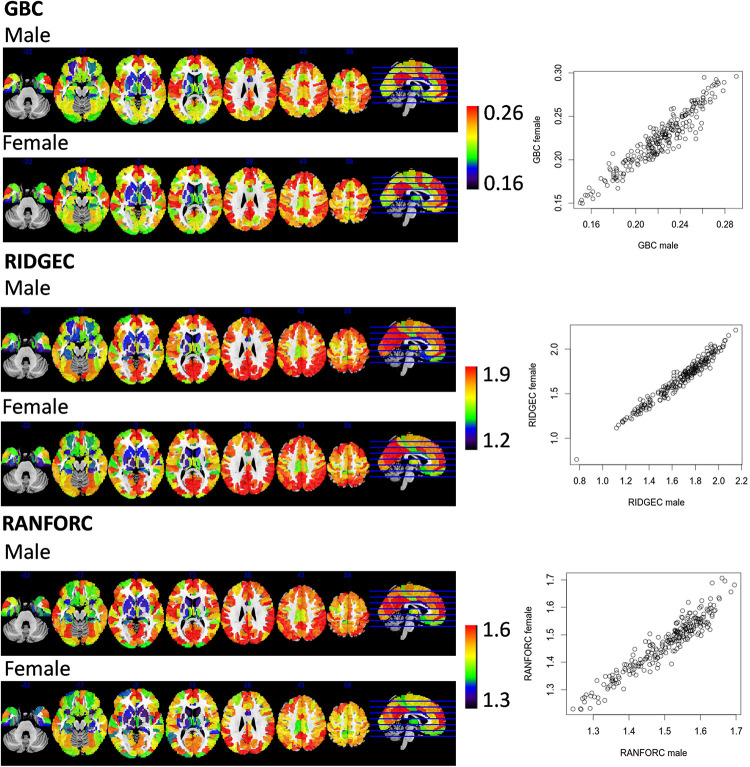
Sex connectivity maps taken from averages of a subsample of 63 males and 63 females matched by age. Scatterplots show, for each one of the 246 regions of the atlas, their averaged scores in males and females. All connectivity values were previously Fisher transformed into z-scores.

**FIGURE 6:**
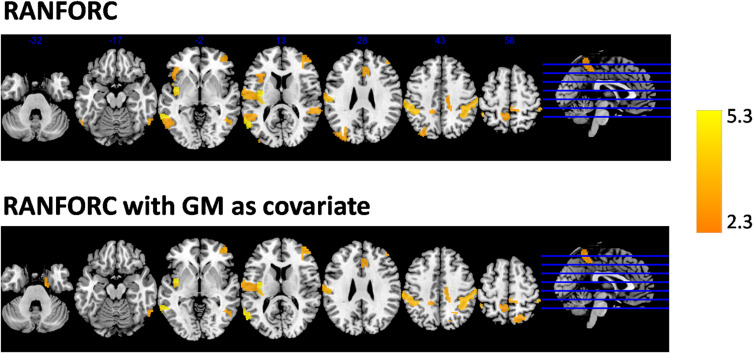
Areas with significantly higher RANFORC in males as reported by tests applied to the regions included in the Brainnetome atlas, with and without considering gray matter partial volumes as covariates in the models, and after FDR correction. No areas of significantly higher female-related connectivity were found with any of the connectivity maps, apart from a single region in the left dorsal cingulate with increased gray matter-corrected GBC in females (not shown in the figure).

Averaged GBC, RIDGEC, and RANFORC maps for the subsample of 63 males and 63 females matched by age are shown in Figure 5. Similarities between both sexes in the three types of connectivity measures are evident from both sex maps and related scatterplots. Indeed, when *t*-tests were run at the region level, none of the areas of the template showed sex differences for the GBC and RIDGEC measures. In contrast, however, the RANFORC revealed a differential pattern of higher male connectivity in many areas including the temporal cortex, the dorsal cingulate and the supramarginal gyri bilaterally, the left insula, and the right dorsolateral prefrontal cortex (see Figure 6). Yet, these differences were not strong enough to lead to non-overlapping confidence intervals between RANFORC and the other two connectivity measures in most of the significant regions (see Supplementary Figure 1).

When gray matter partial volumes were added as covariates, both GBC and RIDGEC remained without showing significant sex-related patterns, apart from a single region (A24rv_L in left dorsal cingulate) with increased GBC in females (see Supplementary Figure 2). On the other hand, RANFORC results kept on having a similar pattern of increased connectivity in males (see Figure 6), although these included some additions and deletions relative to the non-corrected results (see ROI list in Supplementary Figure 2).

When considering the four thresholded variants of the GBC, we found the same non-significant pattern observed in the GBC maps based on averages of absolute values (i.e., no region showed significant differences between males and females in any of the GBC variants).

### Age-Related Connectivity Patterns

**FIGURE 7:**
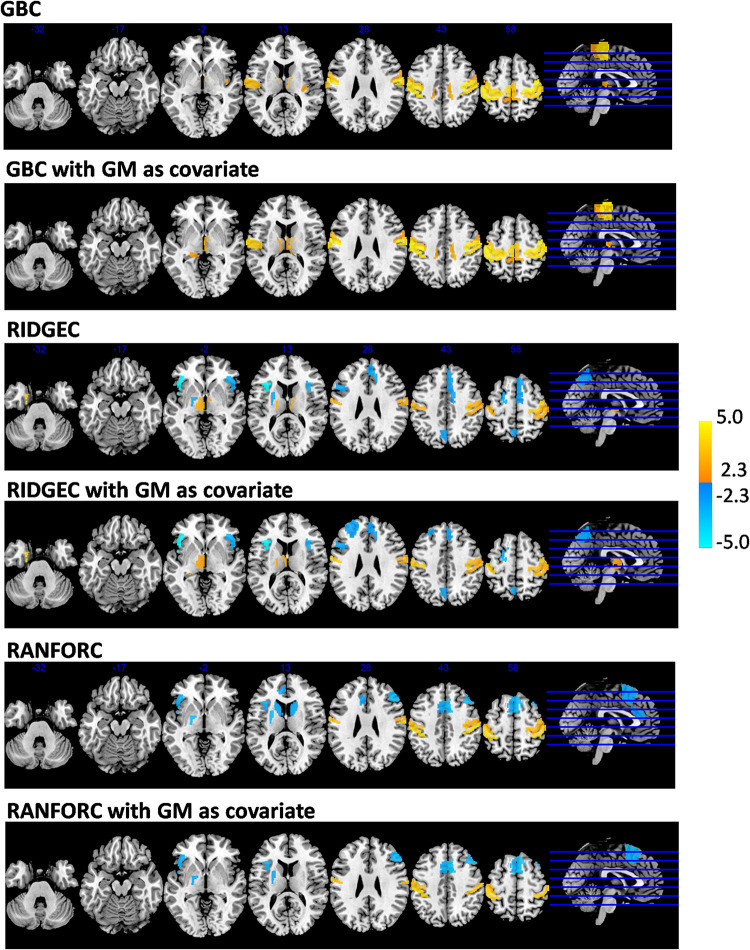
Areas with significant age-related connectivity increases (in yellow) and reductions (in blue) with and without considering gray matter partial volumes as covariates in the models. Although GBC maps only reported on age-related increases, both RIDGEC and RANFORC maps were sensitive enough to find both age-related increases and reductions. Areas shown were significantly related to age after FDR correction for multiple comparisons.

The analysis of age-related connectivity patterns in the sample of 173 healthy individuals led to some common findings but also many differential patterns of age-related connectivity in the GBC, RIDGEC and RANFORC maps. Specifically, linear models taking Fisher-transformed connectivity values as dependent variables, age as the main independent variable, and sex as a nuisance covariate revealed clearly significant age-related results in the three maps (Figure 7). Of relevance, although GBC only showed areas of significantly increased connectivity with age, both RIDGEC and RANFORC were sensitive enough to report on both areas of increased and decreased multivariate connectivity. The only common connectivity pattern shared by all three brain maps was a positive age relation in the bilateral postcentral gyrus, whereas both GBC and RIDGEC also had increased thalamus connectivity with age. On the other hand, shared patterns of age-related disconnectivity in RIDGEC and RANFORC included decreased connectivity in the bilateral anterior insula, medial frontal cortex, and left putamen. In contrast, differential patterns involved age increased GBC connectivity in bilateral paracentral lobules and right posterior insula, age decreased connectivity in RIDGEC in the left precuneus, and age reductions in RANFORC in both caudates. However, not all differential age-related patterns of connectivity were strong enough to elicit non-overlapping confidence intervals between connectivity methods (see Supplementary Figure 1).

Gray matter-corrected findings in GBC were very similar to those of non-corrected images, only including areas of increased connectivity with age (see Figure 7). RIDGEC results were also similar between corrected and non-corrected maps, although the former included four deleted and two new significant ROIs (see list in Supplementary Figure 2). Finally, correction for gray matter partial volumes in RANFORC led to a net, although not severe, reduction in significant areas (5 out of 13 ROIs; see Figure 7 and list in Supplementary Figure 2).

When considering the four thresholded variants of the GBC, we found a similar pattern to that observed in the GBC maps based on absolute correlations, with positive associations between age and GBC. However, there were, as well, few differences. These differences mainly included a small number of ROIs becoming non-significant with the thresholding and a single ROI of the template (A20rv_L, located in the left fusiform gyrus) showing a negative relation with age. A comparative list of significant regions between all versions of the GBC is provided in Supplementary Figure 3.

## Discussion

It is relevant that group averaged maps for the two regularized multivariate connectivity methods proposed here have had similar patterns to those shown by maps based on simpler bivariate correlations (i.e., GBC maps). To some degree, this is not unexpected, as they all deal with similar information, and, to some extent, it points to the absence of major flaws in the methods proposed. On the other hand, it is also important to keep in mind that bivariate and multivariate approaches are exploring different aspects of brain connectivity. A multivariate model, by construction, is bound to be potentially affected by any change in any of the variables (brain regions) considered, whereas the simpler approach based on averaging correlations followed by the GBC is more likely to attenuate them (i.e., individual changes in pairwise correlations will more likely go unnoticed after averaging).

This sensitivity to changes in single regions could be behind the larger number of age-related patterns revealed by both regularized methods, and especially to their capacity to detect age-related disconnection, almost unnoticed by the GBC maps. The higher number of subcortical patterns shown by the two new methods is in agreement with current studies reporting widespread age-related structural changes in subcortical structures (Tullo et al., 2019; Wang et al., 2019), whereas the presence of both age-related increases and decreases in functional connectivity is more in line with recent work in this area (Sala-Llonch et al., 2015; Ferreira et al., 2016; Chen et al., 2019), although non-neural factors such as age-related physiological changes might partially explain some of the findings (Geerligs et al., 2017). In a similar way, the ability to detect sex-related differences by RFs may be attributed to the increased flexibility provided by the lack of additive and linear constraints. One may argue, though, that all these findings are mainly driven by functional artifacts created by differences in gray matter partial volumes. However, the results of the analyses including partial volumes as covariates have dispelled this concern, as they have not usually led to a substantial reduction in the number of significant regions.

From all these considerations, one may conclude that both regularization methods proposed here are feasible alternatives to the more frequently used dimensionality reduction techniques. Still, some methodological aspects should be carefully considered to appropriately apply regularization in brain connectivity. Most crucially, the degree of regularization should remain invariable through all analyses. That is, the values of all regularization parameters should remain constant for all brain regions and through all individual analyses; otherwise, neither the models fitted nor the connectivity scores derived will be comparable. As mentioned in the methods, setting values for regularization parameters is a tradeoff between variance (i.e., increased flexibility of models with its risk of overfitting) and bias, which is generated by imposing too much regularization and which leads to non-flexible models.

Selecting optimal regularization values for connectivity, though, is not straightforward as these values will depend, among other aspects, on the amount of time points available (i.e., length of fMRI time series) and the degree of filtering applied, as this will modulate temporal autocorrelation and the number of effective degrees of freedom in the data. The presence of temporal dependencies in the data will also preclude (or make difficult) using cross-validation techniques, which are the standard method to fine-tune regularization parameters in prediction problems. On the other hand, the amount of regularization required will also depend on the number of brain regions considered in the analyses (i.e., the number of independent variables in the multivariate models). We run exploratory analyses in a single individual to make the initial selection of values for the regularization parameters. This is a rather heuristic approach, but we expect that after the proposed method is applied to datasets of varying characteristics, a limited range of feasible parameter values will emerge for usage in future studies (as it has previously happened with other relevant imaging parameters such as the size of spatial filters, the threshold values for clustering in task-based fMRI, or the correlation thresholds in graph theory). A positive aspect of the methods proposed is that, provided that models are constrained enough, regularization techniques may successfully deal with *N* >> p situations, which will certainly occur if variables are defined by voxels or edges (although large computing capabilities will be required in these situations).

Finally, researchers who are used to perform analyses with correlation matrices may find it inconvenient to have to perform second-level analyses to uncover information on specific pairwise connections (as this information is not provided by primary outputs from the proposed methods). On the other hand, outcomes from our multivariate analyses may be more easily visualized and interpreted than those provided by correlation matrices and connectivity graphs, especially when the number of regions considered is large. Besides, the number of statistical tests to be performed in any intersubject analysis, and its related problem of multiple comparisons and lack of statistical power, will be greatly reduced if the proposed methods are used (this is a benefit that GBC would also hold).

In summary, we propose regularization as a suitable alternative to dimensionality reduction for developing multivariate measures of functional connectivity. Although the two methods proposed (RIDGEC and RANFORC) share some similarities with the much simpler GBC method (based on averages of bivariate correlations), their multivariate nature provides greater sensitivity in detecting age-related connectivity patterns. Furthermore, the greater flexibility offered by the RF algorithm allows for detecting sex-specific differences.

## Data Availability Statement

The raw data supporting the conclusions of this article will be made available by the authors, without undue reservation.

## Ethics Statement

All the study procedures had been previously approved by the local research ethical committee (Comité de Ética de la Investigación (CEI) de FIDMAG Hermanas Hospitalaria). The patients/participants provided their written informed consent to participate in this study.

## Author Contributions

RS, SS, ElV, EdV, EP-C, and AV design of the study. NV, BG-R, EJ, SS, ElV, EdV, and EP-C recruitment of the subjects. NV, BG-R, and EJ acquisition of MR images. EC-R image preprocessing and quality control. RS and EC-R fitting and analysis of statistical models. RS, ElV, EdV, EP-C, and AV manuscript preparation. All authors contributed to the article and approved the submitted version.

## Conflict of Interest

EdV has received grants and served as consultant, advisor or CME speaker for the following entities: AB-Biotics, Abbott, Allergan, Angelini, AstraZeneca, Bristol-Myers Squibb, Dainippon Sumitomo Pharma, Farmindustria, Ferrer, Forest Research Institute, Gedeon Richter, GlaxoSmithKline, Janssen, Lundbeck, Otsuka, Pfizer, Roche, SAGE, Sanofi-Aventis, Servier, Shire, Sunovion, Takeda, the Brain and Behavior Foundation, the Spanish Ministry of Science and Innovation (CIBERSAM), the EU Horizon 2020, and the Stanley Medical Research Institute. The remaining authors declare that the research was conducted in the absence of any commercial or financial relationships that could be construed as a potential conflict of interest.
