# Supplementary material for: Multivariate Brain Functional Connectivity Through Regularized Estimators
Source: Front Neurosci. 2020 Dec 8;14:569540. doi: 10.3389/fnins.2020.569540 (PMC7753183; doi:10.3389/fnins.2020.569540)
Supplement: Supplementary file 1 [file Data_Sheet_1.DOCX]

A


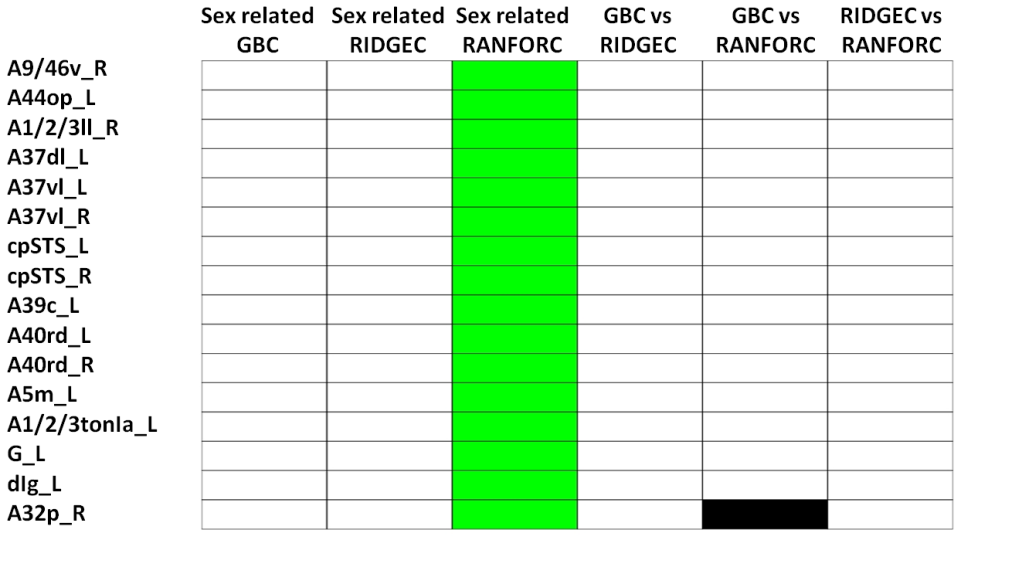


B

**
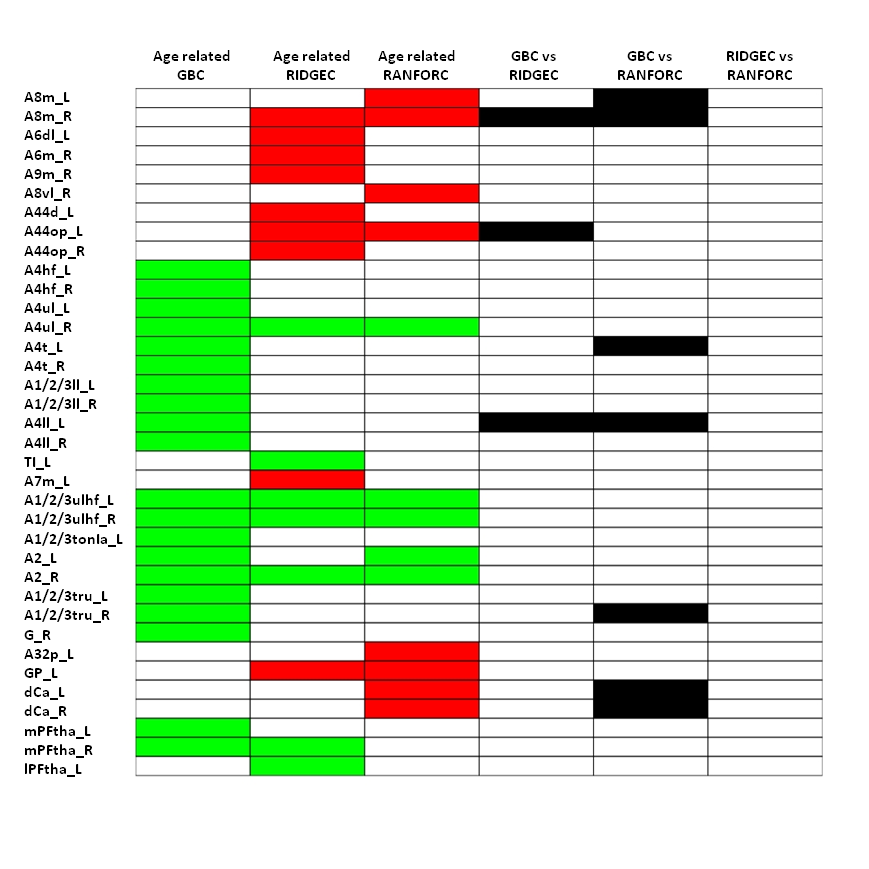
**

**Supplementary Figure 1**: Regions of interest, as coded in the Brainnetome Atlas, where statistically significant patterns related to gender (A) and age (B) were found for the GBC (column 1), RIDGEC (column 2) or RANFORC (column 3). Positive associations are marked in green and negative relations in red. Columns 4-6 highlight those regions where differential patterns between connectivity measures were strong enough to elicit non-overlapping confidence intervals in the estimated parameters from models using standardized values. Such information, though, should be taken cautiously as overlapping confidence intervals are not necessarily equivalent to non-significant differences (i.e. considering the rule of non overlapping confidence intervals as proof of significance may be overconservative).
