# Supplementary material for: Multivariate Brain Functional Connectivity Through Regularized Estimators
Source: Front Neurosci. 2020 Dec 8;14:569540. doi: 10.3389/fnins.2020.569540 (PMC7753183; doi:10.3389/fnins.2020.569540)
Supplement: Supplementary file 2 [file Data_Sheet_2.DOCX]

A


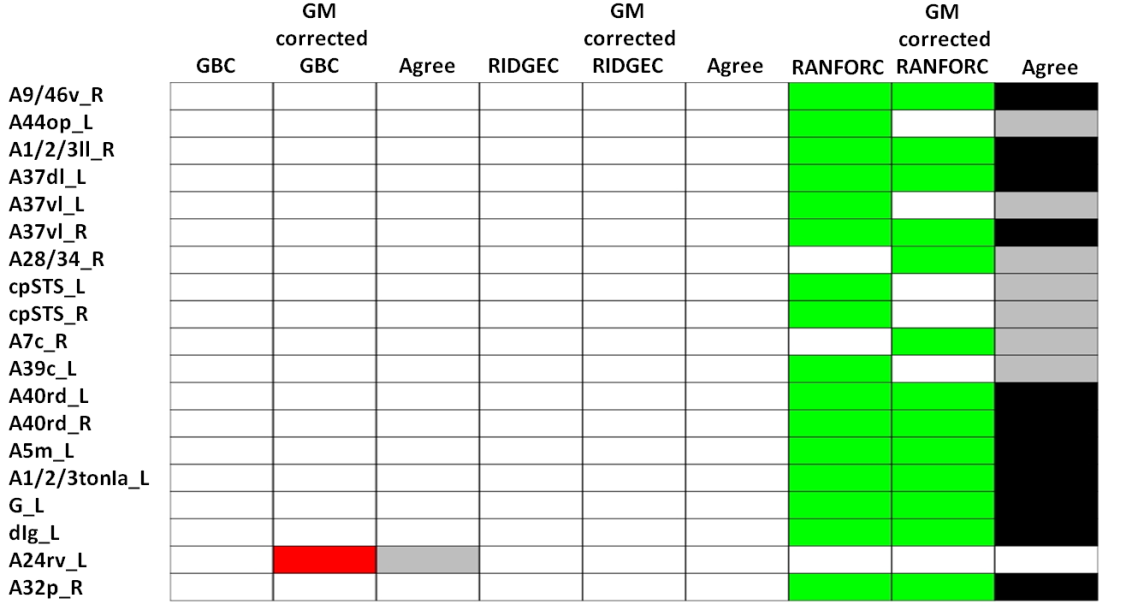


B

**
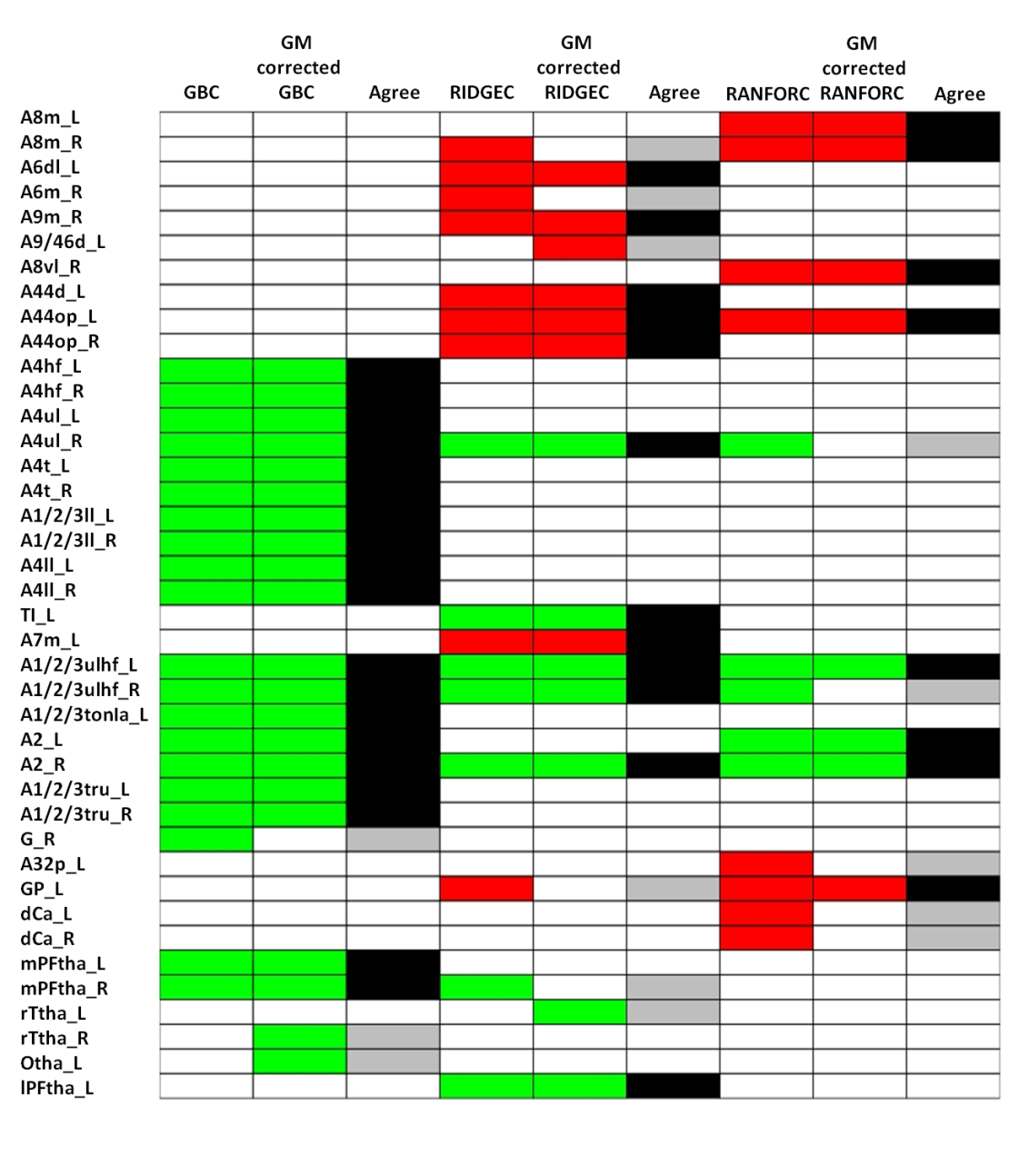
**

**Supplementary Figure 2**: Regions of interest, as coded in the Brainnetome Atlas, where statistically significant patterns related to gender (A) and age (B) were found for any of the three connectivity measures with or without considering grey matter partial volumes as covariates in models (GM corrected). Positive associations are marked in green and negative relations in red. After each pair of columns a third column (Agree) highlights in black the cases were the region is significant with and without GM correction.
