# Supplementary material for: Multivariate Brain Functional Connectivity Through Regularized Estimators
Source: Front Neurosci. 2020 Dec 8;14:569540. doi: 10.3389/fnins.2020.569540 (PMC7753183; doi:10.3389/fnins.2020.569540)
Supplement: Supplementary file 3 [file Data_Sheet_3.DOCX]

**
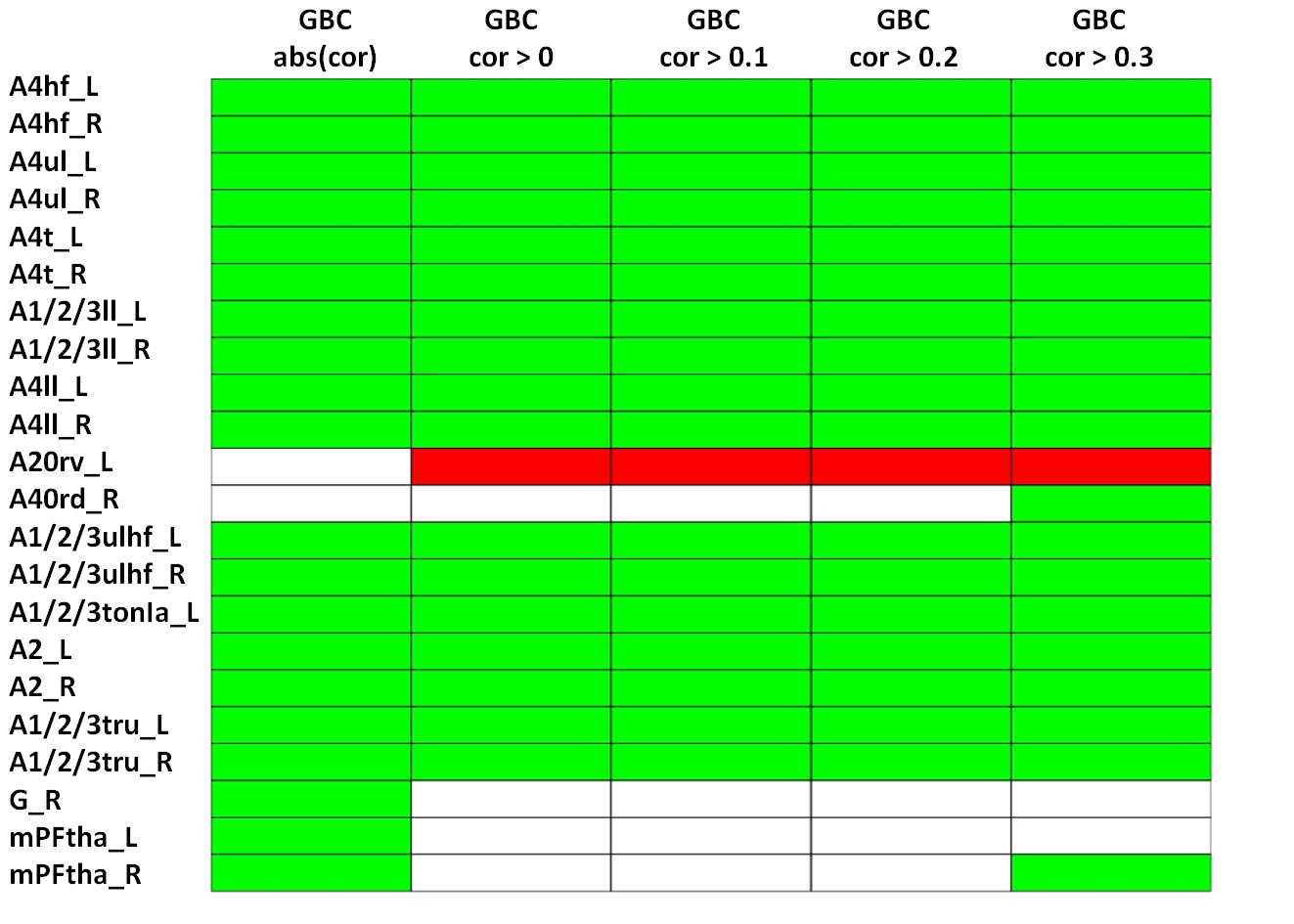
**

**Supplementary Figure 3**: Regions of interest, as coded in the Brainnetome Atlas, where statistically significant patterns related to age were found with any of the GBC variants: GBC based on averaged absolute values of correlations (abs(cor)), GBC based on positive correlations (cor > 0), and on correlations above 0.1, 0.2 and 0.3 (cor > 0.1, cor > 0.2, cor > 0.3). Positive associations are marked in green and negative relations in red.
